# Distinctive coordination behavior of a pyrazole imine-oxime compound towards Co(II) and Ni(II)

**DOI:** 10.1016/j.heliyon.2019.e01623

**Published:** 2019-05-10

**Authors:** Samik Gupta, M. Fátima C. Guedes da Silva, Armando J.L. Pombeiro

**Affiliations:** a Centro de Química Estrutural, Complexo I, Instituto Superior Técnico, Universidade de Lisboa, Av. Rovisco Pais, 1049-001, Lisboa, Portugal; b Department of Chemistry, Sambhu Nath College, Labpur, Birbhum, West Bengal, PIN-731303, India

**Keywords:** Inorganic chemistry

## Abstract

The polytopic Schiff base 5-methyl-1H-pyrazole-3-carboxylic acid 2-(hydroxyimino-1-methyl-propylidene)-hydrazide (H_2_L)was synthesized by the condensation of 5-methyl pyrazole-3-carbohydrazide and 3-(hydroxyimino)butan-2-one and its coordination ability was tested against cobalt (II) and nickel (II) nitrates. The ligand exhibited two different binding modes to form a unique binuclear triply bridged Co(III) cationic complex [Co_2_(1κ*N*^2^:2κ*N*^2^-L) (1κ*N*^3^:2κ*O*^1^-HL)_2_](NO_3_)_2_ (**1**). With the Ni(II) precursor, H_2_L was hydrolyzed to N′,N˝-butane-2,3-diylidenebis (5-methyl-1*H*-pyrazole-3-carbohydrazide) (H_2_L^1^) which bound the metal cation in a tetradentate N_3_O_1_ fashion leading to the neutral square planar complex [Ni(κ*N*^3^*O*^1^-L^1^)]·MeOH (**2**·MeOH). Complexes **1** and **2** were characterized by IR, NMR, UV-Vis and single crystal X-ray crystallography. The probable mechanism for the Ni(II) mediated transformation of H_2_L into H_2_L^1^ has been investigated by ESI-MS.

## 1 Introduction

The coordination chemistry of oximes is versatile because they are used abundantly as complexing agents in the isolation, separation and extraction of various metal ions [1, 2, 3, 4, 5, 6, 7, 8, 9, 10, 11, 12, 13]. The H-bonding pattern and packing of oxime based complexes are also interesting and leads to remarkable optical properties [14, 15]. Moreover, these complexes are biologically significant as they are found to serve as models for biosystems such as vitamin B12 [16] and as myocardial perfusion imaging agents [17]. Hydroxamate complexes can also display a high antitumor activity [18, 19, 20]. Oximes are potentially ambidentate [3, 5] as they can bind through either or both the N [10, 11, 12, 13] or the O [21]atoms. The vast literature on structural studies of oxime complexes reveals some interesting features of its coordination behavior. In the majority of the cases only the nitrogen atom binds to the metal centre, although there are a few examples where both the nitrogen and oxygen atoms of oximato group take part in coordination [22, 23] and even form1,2 (N,O) oximato-bridged extended networks [6, 7, 8, 9]. Furthermore, studies of metal mediated reactions involving oxime compounds have gained impetus in the past decade providing facile techniques for a wide range of organic syntheses.

Our group has been involved in the reaction of oximes [24, 25, 26, 27, 28, 29, 30, 31, 32] with metal bound nitriles. Ni(II)-ketoxime mediated transformations of nitriles or/and phthalonitriles were achieved, affording various nickel-ligated species including (i) symmetrical imidoylamidines (1,3,5-triazapentadienes) [24], (ii) phthalocyanines [25] and (iii) unsymmetrical imidoylamidines with imino-isoindolinone moieties [26]. Ni(II) mediated nitrosation of oximes containing α-CH_2_ groups have also been studied [27].

We now report the synthesis of a Schiff base (H_2_L) by the condensation of 5-methyl 3-pyrazole carbohydrazide and 3-(hydroxyimino) butan-2-one. In the presence of a cobalt salt, the deprotonated form of H_2_L acts as a tetradentate donor for the metal cation and produces a triply bridged binuclear complex (a unique structure where the same ligand forms two azo-oxo bridge and one diazo bridge between two Co(III) ions). Triple bridged binuclear cores in Co complexes are rare. A few carbon monoxide bridged and hydride bridged species are known. There is also one report of a triply bridged thiolate complex [33] but oxime based triple bridge has not been reported earlier to the best of our knowledge. With a nickel salt, H_2_L undergoes an in-situ hydrolytic transformation to produce a mononuclear neutral square planar complex of a new di-imine ligand. The two complexes were characterized by IR, NMR, UV-Vis and single-crystal X-ray diffraction. The mechanism of the Ni(II) mediated ligand transformation has been investigated by ESI-MS spectroscopy. We propose that the Schiff base H_2_L binds Ni(II) in such a way so as to promote its hydrolysis and rearrangement, ultimately resulting in the formation of a more thermodynamically stable complex.

## 2 Experimental

### 2.1 Materials and methods

5-methyl 3-pyrazole carbohydrazide was prepared according to a literature process [34]. 3-(hydroxyimino) butan-2-one, Co(NO_3_)_2_.6H_2_O and Ni(NO_3_)_2_.6H_2_O were purchased from Aldrich and used as received. Infrared spectra (4000–400 cm^−1^) were recorded on a Nicolet Impact 400D or a BIO-RAD FTS 3000 MX spectrophotometer instrument in KBr pellets; wave numbers are in cm^−1^; abbreviations: vs, very strong; s, strong; ms, medium strong; m, medium; br, broad. UV-Vis spectra were recorded in 10^−4^ M MeOH solutions of the complexes with a Perkin Elmer instrument (Lambda 35). ^1^H NMR spectra were recorded at ambient temperature on a Bruker Avance II 300 (Ultra Shield Magnet) spectrometer operating at 300.130 MHz. The chemical shifts (δ) are reported in ppm using tetramethyl silane as the internal reference. Electrospray mass spectra (ESI-MS) were run with an ion-trap instrument (Varian 500-MS LC Ion Trap Mass Spectrometer) equipped with an electrospray ion source. For electrospray ionization, the drying gas and flow rate were optimized according to the particular sample with 35 p.s.i. nebulizer pressure. Scanning was performed from *m*/*z* 50 to 1000 in CH_3_OH solution. The compounds were studied in both positive and negative modes (capillary voltage = 80–105 V). Other abbreviations used (NMR): s: singlet, T1: type-I binding mode as HL^−^, T2: type-II binding mode as L^2−^.

### 2.2 Synthesis of 5-methyl-1H-pyrazole-3-carboxylic acid (2-hydroxyimino-1-methyl-propylidene)-hydrazide (H_2_L)

1.4 g (10 mmol) of 5-methyl-3-pyrazole carbohydrazide was dissolved in 50 mL ethanol. A 25 mL ethanolic solution of 1.01 g (10 mmol) of 3-(hydroxyimino) butan-2-one was added to that and the mixture was refluxed for 4 h at 100 °C with continuous stirring. Within this time a white precipitate appeared. The solution was cooled to room temperature and kept standing overnight. The precipitate thus formed was filtered off washed with ethanol and ether, and dried over fused CaCl_2_.

Yield: 1.78 mg (80%) white solid soluble in hot EtOH, MeOH, etc.Anal. Calc. for C_9_N_5_O_2_H_13_ (F.W = 223.10): C, 48.40; H, 5.87; N, 31.38. Found: C, 48.27; H, 5.66; N, 31.21. IR (KBr, selected bands, cm^−1^): 3380 s (νNH), 3201vs (νOH), 1675s (νC=O), 1605s (νC=N), 1541 s (νC=C), ^1^H NMR (300.13 MHz, DMSO-D_6_), δ: 2.014 (s, 3H, CH_3_C=N–C(CH_3_)), 2.13 (s, 3H, CH_3_C=N–OH), 2.50 (s, 3H, CH_3_-pyrazole ring), 6.56 (s, 1H, pzC4-H) 8.2 (s, 1H, NH–C=O), 10.2 (C=N–OH), 11.63 (s, 1H, pzN-H).

### 2.3 Synthesis of [Co_2_(1κ*N*^2^:2κ*N*^2^-L)(1κ*N*^3^:2κ*O*^1^-HL)_2_](NO_3_)_2_ (**1**)

To 50 mg (0.224 mmol) of H_2_L, 20 mL methanol was added. The suspension was refluxed for 15 minutes at 80 °C to allow total dissolution of H_2_L. Then, 10 mL of a methanolic solution of Co(NO_3_)_2_.6H_2_O (43 mg, 0.149 mmol) was added. Five drops of water were also added. A dark red solution resulted immediately. The reaction mixture was then refluxed for 3 h at 100 °C during which the color of the solution darkened. After cooling and filtering, the mixture was kept for slow evaporation and crystals of **1** suitable for X-ray diffraction were obtained after almost complete evaporation of solvent.

Yield: 0.054 g (80%) white solid soluble in hot EtOH, MeOH etc. Anal. Calc. for C_27_H_36_ N_17_O_12_ Co_2_ (F.W = 908.13): C, 35.67; H, 3.99; N, 26.21. Found: C, 35.43; H, 3.66; N, 25.94. MS (ESI^+^): *m*/*z*: 782.2 (M^+^) IR (KBr, selected bands, cm^−1^): 3410s (νNH), 1654s (νC=O), 1618s (νC=N), 1560s (νC=C), 1472, 1315, 1050 (ms, νN-Npz), 807 (νNO_3_^−^).^1^H NMR (300.13 MHz, DMSO-D_6_), δ: 2.02 (s, 6H, CH_3_C=N–C(CH_3_)) (T1), 2.16 (s, 3H, CH_3_C=N–C(CH_3_)) (T2), 2.34 (s, 9H, CH_3_C=N–OH) (T1 and T2), 2.51 (s, 9H, CH_3_-pz ring) (T1 and T2), 6.87 (s, 2H, pzC_4_-H) (T1), 7.14 (s, 1H, pzC_4_-H) (T2), 7.97 (s, 2H, NH–C=O) (T1), 13.5 (s, 2H, pzN-H) (T1), 14.12 (s, 1H, pzN-H) (T2). UV-Vis (MeOH, *λ*_max_, nm (ε, L M^−1^ cm^−1^): no prominent peaks in the visible or UV region.

### 2.4 Synthesis of [Ni(κ*N*^3^*O*^1^-L^1^)]·MeOH (**2**·MeOH)

50 mg (0.224 mmol) of H_2_L were mixed with 25 mL methanol and the mixture refluxed for 15 min until complete dissolution. 10 mL of a methanolic solution of Ni(NO_3_)_2_.6H_2_O (130 mg, 0.448 mmol) were then added. Upon the addition of five drops of water no change of the green color of the solution was observed. The reaction mixture was then refluxed at 100 °C for 4 hrs and during this procedure the solution gradually changed to yellow with precipitation of an orange compound. The solid was filtered off, dissolved in hot MeOH and the solvent allowed for slow evaporation. X-ray quality crystals appeared within 24 h.

Yield: 0.032 g (70%) orange solid soluble in hot EtOH, MeOH etc. Anal. Calc. for C_15_H_20_N_8_O_3_Ni (F.W = 418.10): C, 43.05; H, 4.82; N, 26.79. Found: C, 42.95; H, 4.79; N, 26.60. MS (ESI^+^): *m*/*z*: 387.1 (M-MeOH + H^+^)^+^ IR (KBr, selected bands, cm^−1^): 3380s (νNH), 3200 (νOH), 1685s (νC=O), 1629s (νC=N), 1535s (νC=C), 1223s (νC-O^-^) 1056 (ms, νN-Npz). NMR (300.13 MHz, DMSO-D_6_), δ: 2.24 (s, 6H, CH_3_–C=N-), 2.30 (s, 6H, CH_3_-pz), 6.4 and 6.5 (s, 2H, pzC5-H), 12.84 and 13.9 (s, 2H, pzN-H). UV-vis [MeOH, *λ*_max_, nm (ε, L M^−1^ cm^−1^]: 515 (4300), 356 (13500), 272 (34500).

### 2.5 Crystallographic measurements

**Fig. 1:**
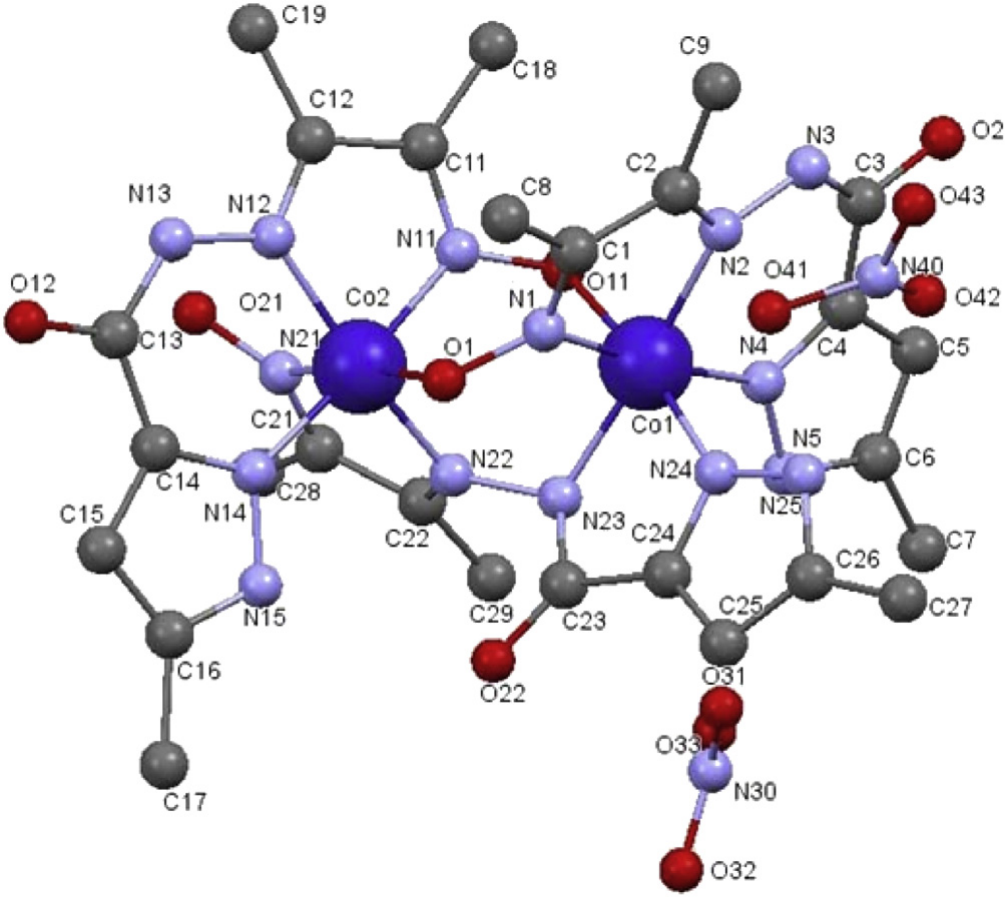
Molecular structure of complex **1** with atom numbering scheme. Hydrogen atoms and nitrate counter-ions were excluded for clarity. Selected bond distances (Ǻ) and angles (°): Co1–O11 1.917 (2), Co1–N1 1.893 (3), Co1–N2 1.932 (3), Co1–N4 1.926 (3), Co1–N23 1.945 (2), Co1–N24 1.906 (3), Co2–O1 1.976 (2), Co2–N11 1.879 (3), Co2–N12 1.939 (3), Co2–N14 1.908 (3), Co2–N21 1.899 (3), Co2–N22 1.873 (3), O2–C3 1.284 (4), O12–C13 1.229 (5), O22–C23 1.224 (4); O11–Co1–N1 91.87 (11), O11–Co1–N2 89.84 (11), O11–Co1–N4 89.34 (11), O11–Co1–N23 92.01 (11), O11–Co1–N24 172.41 (11), N1–Co1–N2 81.27 (12), N1–Co1–N4 172.29 (11), N1–Co1–N23 91.77 (12), N1–Co1–N24 88.51 (13), N2–Co1–N4 91.12 (12), N2–Co1–N23 172.86 (12), N2–Co1–N24 97.71 (12), N4–Co1–N23 95.80 (12), N4–Co1–N24 91.29 (13), N23–Co1–N24 80.40 (12), O1–Co2–N11 91.40 (11), N14–Co2–N22 96.19 (13), N21–Co2–N22 82.09 (12). Hydrogen bonds [*d* (D**···**A) Å, ∠(DHA) °]: N5–H5A⋅⋅⋅O31 2.885 (5), 152, N15–H15⋅⋅⋅O22 2.883 (4), 139, O21–H21⋅⋅⋅N12 2.811 (5), 133, O21–H21⋅⋅⋅N13 3.314 (5), 149, N25–H25A⋅⋅⋅O12^*i*^ 2.969 (5), 120, N25–H25A⋅⋅⋅O42 2.767 (7), 158. Symmetry operation *i*) -1+x,y,z.

**Fig. 2:**
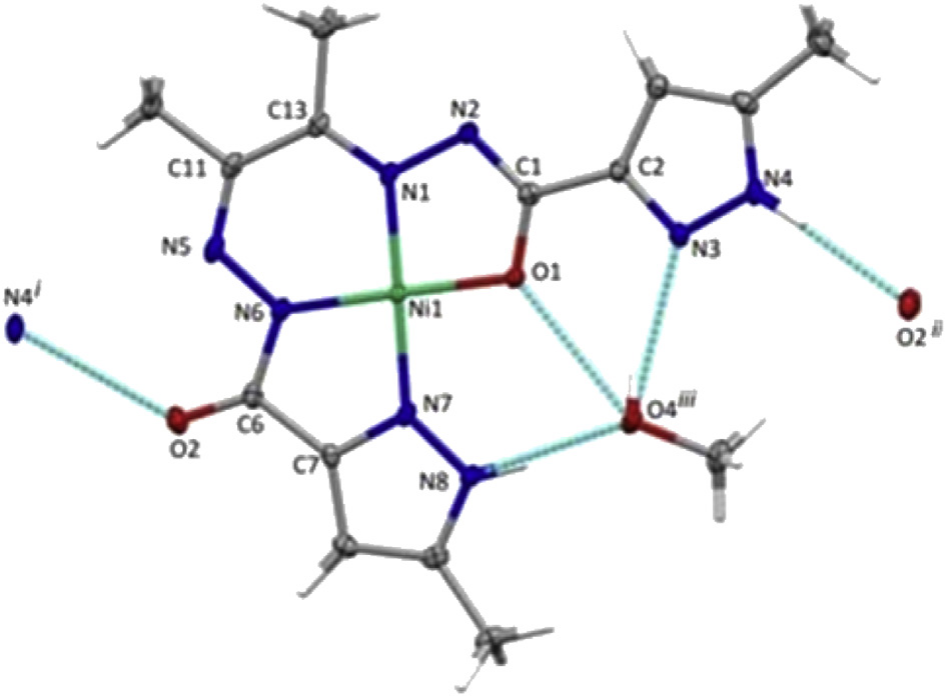
Molecular structure of **2** with atom numbering scheme and hydrogen bond interactions. Ellipsoids are drawn at 30% probability. Selected bond distances (Ǻ) and angles (°): Ni1–O1, 1.8306 (19), Ni1–N1 1.820 (2), Ni1–N6 1.822 (2), Ni1–N7 1.861 (2), O1–C1 1.299 (3), O2–C6 1.227 (3), N2–C1 1.312 (4), N6–C6 1.380 (4); O1–Ni1–N1 85.12 (9), O1–Ni1–N6 177.16 (10), O1–Ni1–N7 95.64 (9), N1–Ni1–N6 95.25 (10), N1–Ni1–N7 179.09 (10), N6–Ni1–N7 84.01 (10). Hydrogen bonds [*d* (D**···**A)Å, ∠(DHA) °]: O4–H4⋅⋅⋅O1 2.805(3), 123(3), O4–H4⋅⋅⋅N3 2.969(3), 166(3), N4–H4N⋅⋅⋅O2 2.896(3), 158(3), N4–H4⋅⋅⋅N5 3.243(3), 133(3), N8–H8N⋅⋅⋅O4 2.724(3), 161(3). Symmetry operations to generate equivalent atoms: i) x,1/2-y,-1/2 + z; ii) x,1/2-y,1/2 + z; iii) 1 + x,1/2-y, 1/2 + z.

Crystals were immersed in cryo-oil, mounted in a Nylon loop and measured at a temperature of 150 K. Intensity data were collected using a Bruker AXS-KAPPA APEX II diffractometer with graphite monochromatic Mo-Kα (λ = 0.71073 Å) radiation. Data were collected using omega scans of 0.5° per frame and full sphere of data were obtained. Cell parameters were retrieved using Bruker SMART software and refined using Bruker SAINT [35] on all the observed reflections. Absorption corrections were applied using SADABS [35]. Structures were solved by direct methods by using the SHELXS–97 package [36] and refined with SHELXL–97 [36]. Calculations were performed using the WinGX System–Version 1.80.03 [37]. All hydrogen atoms were inserted in calculated positions. There were disordered solvents present in the structures of complex **1**. Since no obvious major site occupations were found for those molecules, it was not possible to model them. PLATON/SQUEEZE [38] was used to correct the data and potential volume of 405 Å^3^ was found with 199 electrons per unit cell worth of scattering. Least square refinements with anisotropic thermal motion parameters for all the non-hydrogen atoms and isotropic for most of the remaining atoms were employed. Crystallographic details are listed in Table1 and selected bond distances and angles in the legends of Figs. 1 and 2. CCDC 1857604 (**1**) and 1857605 (**2**) contain the supplementary crystallographic data for this paper. These data can be obtained free of charge from The Cambridge Crystallographic Data Centre via www.ccdc.cam.ac.uk/data_request/cif.

**Table 1:** Crystallographic data for compounds **1** and **2**.

Compound	1	2
Formula moiety	C_27_H_36_Co_2_N_15_O_6_, 2NO_3_	C_14_H_16_N_8_NiO_2_,CH_3_OH
Formula Weight	908.59	419.10
Crystal System	Triclinic	Monoclinic
Space group	P -1	P 2_1_/c
*a*(Å)	11.6445 (6)	7.5086 (4)
*b*(Å)	12.2598 (7)	10.9330 (5)
*c*(Å)	16.6466 (4)	22.5108 (11)
α(°)	74.786 (3)	90
β(°)	81.925 (2)	92.788 (3)
γ(°)	70.448 (3)	90
V [Å^3^]	2157.31 (17)	1845.76 (16)
Z	2	4
ρcalc (Mg/m^3^)	1.399	1.508
*μ*(Mo Kα) (mm^−1^)	0.841	1.086
F (000)	934	872
Refls collected/observed/unique	25962/7811/5394	14585/3775/2741
R_int_	0.0399	0.056 5
R_1_, wR_2_ (I ≤ 2σ)^a^	0.0487, 0.1387	0.0385, 0.0890
R_1_, wR_2_ (all data)	0.0738, 0.1502	0.0656, 0.1012
GOF	1.018	0.943

a R_1_ = ∑||*F*o| – |*F*c||/∑|*F*o|. wR_2_ = [∑[w (*F*o^2^ – *F*c^2^)2]/∑[*w* (*F*o^2^)^2^]]^1/2^.

## 3 Results and discussions

### 3.1 Syntheses

**Fig. 3:**
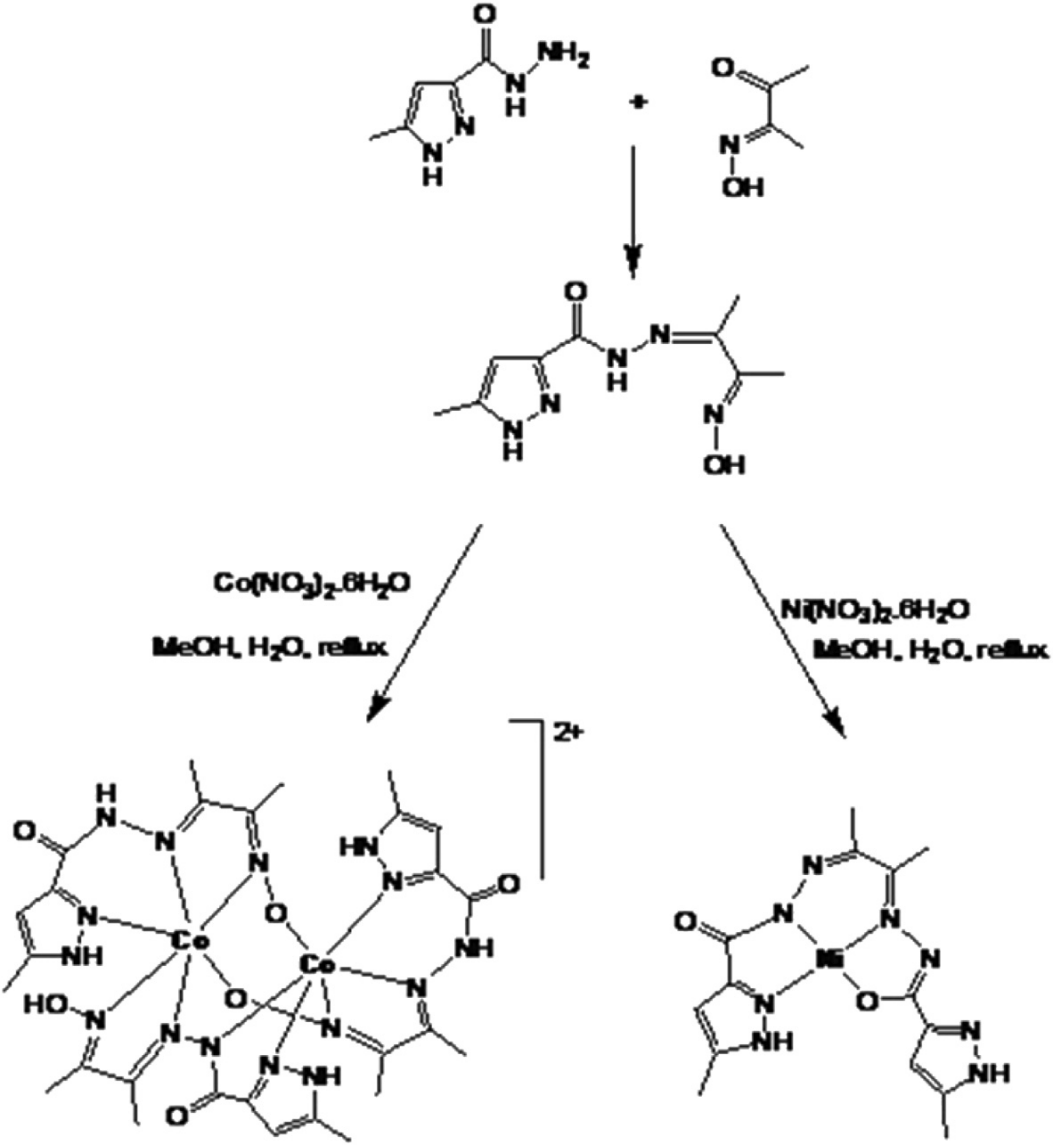
Reaction scheme for the preparation of complexes **1** and **2**.

Pro-ligand H_2_L has been prepared by the simple condensation of 5-methyl 3-pyrazole carbohydrazide and 3-(hydroxyimino) butan-2-one in 1:1 proportion in ethanolic solution. Complex **1** was prepared by refluxing H_2_L and Co(NO_3_)_2_.6H_2_O in methanolic solution. Complex **2** on the other hand was prepared by refluxing H_2_L and Ni(NO_3_)_2_.6H_2_O in a methanolic solution A metal mediated transformation of H_2_L was brought about here leading to the formation of a diimine H_2_L^1^ and eventually the square planar complex **2** was formed (Fig. 3). Complexes **1** and **2** were characterized by IR, NMR, UV-Vis and single crystal X-ray crystallography.

### 3.2 Molecular structures of complexes **1** and **2**

**Fig. 4:**
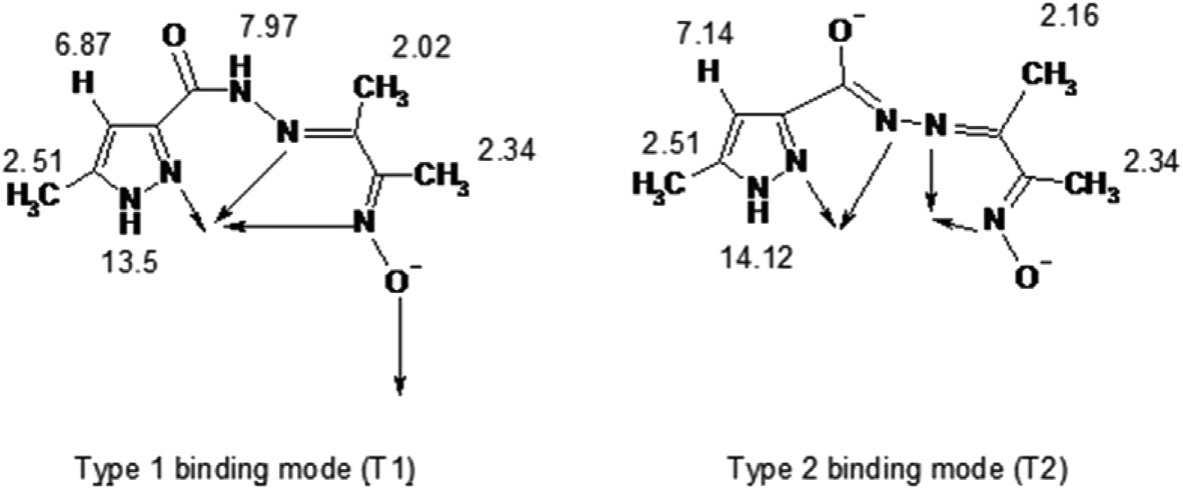
^1^H-NMR signals(δ) for the complex **1**.

The asymmetric unit of [Co_2_(1κ*N*^2^:2κ*N*^2^-L) (1κ*N*^3^:2κ*O*^1^-HL)_2_](NO_3_)_2_ (**1**) comprises one complex molecule and two nitrate counter ions (Fig. 1). This binuclear cobalt (III) complex contains the metal cations in slightly distorted N_5_O_1_ octahedral environments with no sharing of vertices or edges. The three organic moieties act as chelating and bridging entities standing as a tetradentate all-*N* dianionic ligand in a 1κ*N*^2^:2κ*N*^2^ fashion or as *N*_3_*O*_1_ monoanionic ligands in a 1κ*N*^3^:2κ*O*^1^ mode. In this latter type (T1binding mode in Fig. 4) of coordination the ligands are almost planar and their oxime groups form azo-oxobridges between the metal centres, similar to that exhibited by other oxime ligated binuclear Co complexes [39, 40, 41, 42, 43]. In the former category (T2 binding mode in Fig. 4), however, the ligand is strongly twisted as evidenced by the angle of 66.87° between the mean planes of the two five membered rings Co1–N24–C24–C23–N23 and Co2–N22–C22–C21–N21. Each metal cation is involved in two five-membered CoN_2_C_2_ and one six membered CoN_3_C_2_ metallacycles. Additionally, there are two Co_2_N_3_O_1_ and one Co_2_N_2_O_2_ rings that result from the triply bridged Co(III) centres which, therefore, generate the novel tricyclo binuclear Co core. The distance between the two Co cations is of 3.3763 (7) Å. Among the Co–N bond distances [in the 1.879(3) – 1.945(3) Å range] those involving the N_oxime_ atoms are the shortest. The Co1–O11 length [1.917 (2) Å] is considerably shorter than that of Co2–O1 [1.976 (2) Å] what may be related to higher *trans* effect of the oxime group in the latter case, relative to pyrazole in the former.

Complex **2** (Fig. 2) crystallized in the monoclinic system (space group P2_1_/c) and the asymmetric unit consists of one molecule of the complex and one molecule of methanol. The tetradentate (L^1^)^2−^ ligand coordinates the nickel cation in a N_3_O_1_ fashion by means of the amide oxygen (O1), the pyrazol nitrogen (N7), the azomethine nitrogen (N6) and the diazine nitrogen (N1). The metal cation adopts an almost perfect square planar geometry, sustained by the low value (0.03) of the structural parameter *τ*_4_ = [360° – (α + β)]/141° [44],whose values range from 0.00 for a perfect square pyramid to 1.00 for a perfect tetrahedron, withα and β being the two largest angles in the complex.

**Fig. 5:**
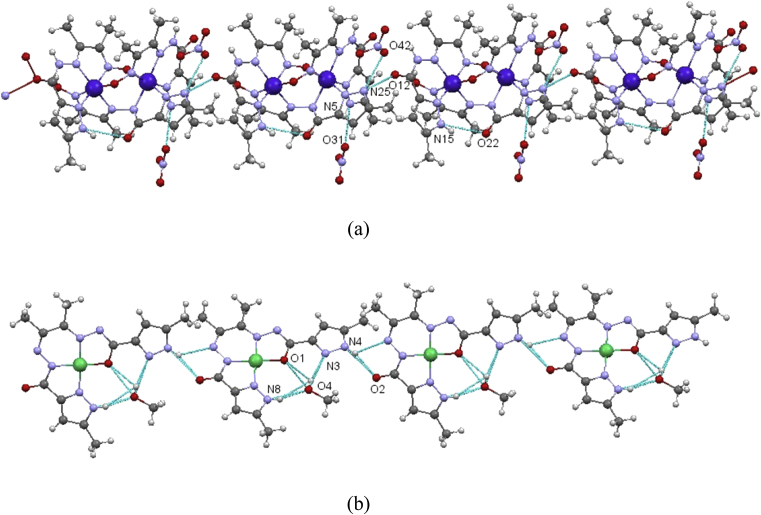
Fragments of the 1D chains which run along the crystallographic *a* axis in complex **1**(a), and along the *c* axis in complex **2**(b).

Both complexes **1** and **2** are involved in relevant non-covalent interactions. The pyrazole N5 and N25 atoms in the crystal lattice of **1** act as H-donors to the nitrate O31, O42 respectively. N15 and N25 also produces H-bonds with O22 and O12 of carbohydrazone portion of the ligand leading to the formation of 1D chain that spread along the crystallographic *a* axis (Fig. 5a). Further stabilization of the structure is also achieved by means of intramolecular medium-strong non-covalent π⋅⋅⋅π interactions, *e.g.* between Co2–N1–C1–C2–N2 and Co2–N11–C11–C12–N12 metalacycles (*centroid*⋅⋅⋅*centroid* distance of 3.501 (2) Å). The molecules of **2** are connected by means of the pyrazole N4 atom which acts as donor not only to the carbonyl O2 atom but also to N5. Additionally, the methanol molecule behaves as donor to O1 and to the pyrazole N3 atom and, simultaneously, as acceptor of the N8-pyrazole hydrogen. Such contacts extend the molecules into infinite 1D chain along the crystallographic *c* axis (Fig. 5b).

### 3.3 Spectroscopic characterization

In the ^1^H-NMR spectra, the chemical shifts observed forcompound H_2_L unambiguously confirm its structure. In particular, the presence of the two highly deshielded singlets at *δ*10.2 and 11.63 for the oxime OH and the pyrazole NH, respectively [45, 46]. In complex **1,** the ligand assumes two different binding modes (Fig. 4) as indicated above.

In Type-I binding mode denoted as T1, ligand (HL^−^) is mononegative (less conjugated structure) and hence the proton signals are less deshielded, while in the Type-2 binding mode, denoted as T2, it is binegative with extended conjugation, the proton signals generally being more deshielded. However no distinct resonances, corresponding to the two different binding modes are exhibited by the methyl group protons of the pyrazole ring and the methyl group associated to oxime functionality (Fig. 4).

The ^1^H-NMR signals of **2** clearly show the changes due to ligand transformation, when compared to those of H_2_L and **1**. The signal for the oxime OH group which is present in the spectra of H_2_L, is absent in **2**. The azomethine proton signal present in both spectra of H_2_L and **1** is also absent in **2**. The single signal at δ11.63 for Pz-NH in the spectra of H_2_L is replaced by two different signals in **2** due to presence of two non equivalent Pz–NH groups. The Pz –NH signal at *δ*12.84 is assigned to the uncoordinated pyrazole ring while that at *δ*13.9 is due to the coordinated pyrazole ring. The pyrazole C–H proton appears at *δ*6.4 and *δ*6.5 for the non-coordinated and coordinated pyrazole rings respectively.

In complex **1**, the absence of the ν(OH) band indicates deprotonation of this group in both binding modes and the higher energy shifts of both the ν(C=N) and ν(C=C) bands are indicative of metal binding [47]. While the ν(C=O) band in **1** shifts to lower energy as compared to that of the pro-ligand (Δν = 21 cm^−1^), this band in **2** suffers a 10 cm^−1^ high energy shift. The non-existence of the ν(OH) band in the spectrum of complex **2** accounts for the absence of the oxime function in the (L^1^)^2−^ ligand.

In MeOH solution, complex **1** does not show any absorption bands in the visible and UV region as was the case with a few other bridged Co(II) complexes [48]. However, complex **2** shows a weak band at 515 nm assigned to the d-d transition in the square planar Ni(II) geometry [49]; the absorption at 356 nm is probably a charge transfer bandand that at higher energy (272 nm) can be assigned to intra-ligand transitions [50].

### 3.4 Proposed mechanism of the Ni(II) mediated reaction

The imino-oxime H_2_L is hydrolytically stable in the presence of Co(II) ions in aqueous MeOH. The oxidation of the metal takes place in-situ and the ligand binds the Co(III) ions keeping its integrity, exhibiting two distinctly different binding modes. The resulting binuclear Co(III) complex**1** was isolated in fairly good yield and fully characterized. However, in the presence of the Ni(II) cation a metal mediated hydrolytic transformation of H_2_L to H_2_L^1^ takes place and the latter entirely fulfills the four coordination positions of the square planar complex [Ni(L^1^)](**2**). The observed transformation is similar to one previously reported by Kelly *et al.* [51].

**Fig. 6:**
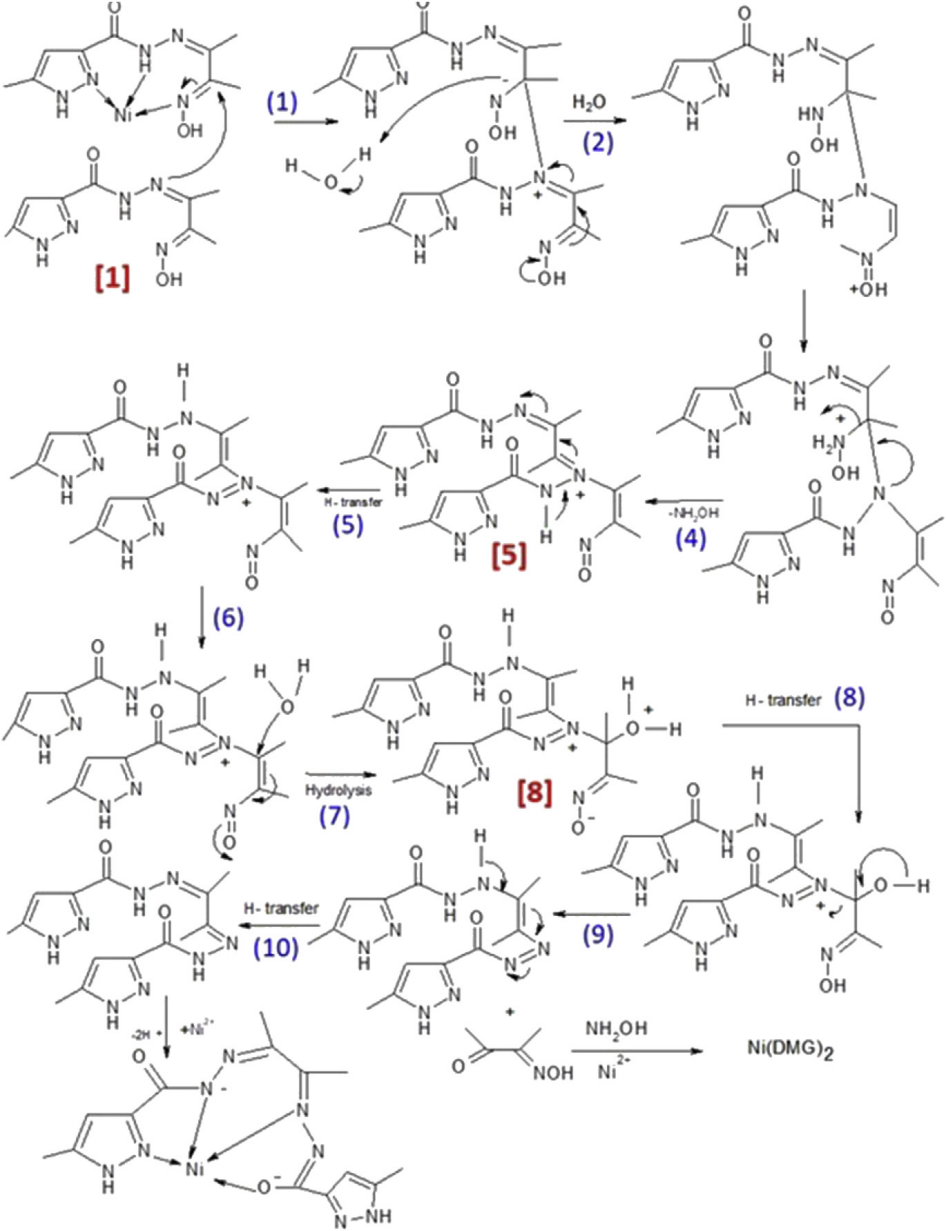
Proposed mechanism for the Ni(II) mediated conversion of the imino-oxime H_2_L to the diimine H_2_L^1^. Reaction steps are indicated in blue and relevant intermediates in red. Steps 5, 8 and 10 involve intermolecular H^+^ transfer.

In order to elucidate the mechanism for our nickel mediated transformation we have monitored the reaction by ESI-MS every 2 h for a period of 6 h. Immediately after the addition of the nickel salt to an aqueous MeOH solution of H_2_L, a peak at *m/z* 503 appeared (100% abundant), probably due to a species resulting from the nucleophilic attack of a H_2_L molecule to another Ni^2+^bound H_2_L centre (Fig. 6, species 1). This entity may undergo rearrangements (Fig. 6, steps 2 and 3) followed by elimination of hydroxyl amine (Fig. 6, step 4) and leading to an intermediate (Fig. 6, species 5) which could be traced as a water adduct (Fig. 6, species 8) at *m/z* 420 after 2 h. Subsequent elimination of dimethyl glyoxime (DMG) (Fig. 6 steps 8 and 9) may lead to the formation of H_2_L^1^ in the reaction medium. The very stable Ni-DMG complex [Ni(DMG)_2_], was detected at *m/z* 262 (also *m/z* 298, M^+^+ 2H_2_O) after 4 h, its abundance growing up to 54 % of the final product. The formation of the diimine species H_2_L^1^ could also be traced by the appearance and gradual increase of the peak at *m/z* 346. The instability of the pro-ligand only in presence of Ni(II) may be driven by a hydrolytic pathway which leads to formation of thermodynamically stable Ni(DMG)_2_ during the hydrolysis process.

## 4 Conclusion

We have investigated the coordination ability of a polytopic imine-oxime compound towards cobalt and nickel cations. While the Co(II) precursor underwent an *in situ* oxidation to Co(III) forming a binuclear complex with novel bridging mode, the Ni(II) eventually triggered a hydrolytic transformation of the imine-oxime ligand to a diimine, which then bound the metal in a tetradentate mode giving rise to a stable square planar Ni(II) complex. A study of the reaction involving this cation by ESI-MS gave evidence for a metal mediated reaction involving a nucleophilic substitution at the oxime moiety of the ligand, conceivably activated by complexation, followed by rearrangement and eventual elimination of diacetyl monoxime.

## Declarations

### Author contribution statement

Samik Gupta, M. Fátima C. Guedes da Silva, Armando J.L. Pombeiro: Conceived and designed the experiments; Performed the experiments; Analyzed and interpreted the data; Contributed reagents, materials, analysis tools or data; Wrote the paper.

### Funding statement

This work was supported by the Foundation for Science and Technology (FCT) (UID/QUI/00100/2013), Portugal.

### Competing interest statement

The authors declare no conflict of interest.

### Additional information

Data associated with this study has been deposited at The Cambridge Crystallographic Data Centre under the accession number CCDC 1857604 (1) and 1857605 (2).
